# Cybervictimization and Depression among Adolescents: Coping Strategies as Mediators

**DOI:** 10.3390/ijerph19073903

**Published:** 2022-03-25

**Authors:** Poh Chua Siah, Xiang Yi Tee, Joanna Tjin Ai Tan, Chee Seng Tan, Komathi Lokithasan, Sew Kim Low, Chin Choo Yap

**Affiliations:** 1 Department of Psychology and Counseling, Universiti Tunku Abdul Rahman, Kampar Campus, Kampar 31900, Perak, Malaysia; yutee2109@1utar.my (X.Y.T.); tcseng@utar.edu.my (C.S.T.); komathil@utar.edu.my (K.L.); milowsk@gmail.com (S.K.L.); 2 Department of Languages and Linguistics, Universiti Tunku Abdul Rahman, Kampar Campus, Kampar 31900, Perak, Malaysia; tanta@utar.edu.my; 3 Department of Psychology, Sunway University, Petaling Jaya 47500, Selangor, Malaysia; gracey@sunway.edu.my

**Keywords:** cybervictimization, coping strategies, depression, adolescents, transactional model of stress and coping theory

## Abstract

Studies have shown the relationships among cybervictimization, coping strategies, and depression, but no study has examined the mechanism that links the three variables. Accordingly, this study used the transactional model of stress and coping theory as a conceptual framework and proposed that coping strategies are mediators for the effects of cybervictimization on depression. A total of 387 adolescents were recruited by using the purposive sampling method. The results showed that cybervictimization is not directly associated with depression. All the coping strategies are found to be associated with cybervictimization, but only the avoidant coping strategy is the statistical mediator for the effects of cybervictimization on depression. This study’s findings suggest that the transactional model of stress and coping theory may provide a framework in the area of cyberbullying and recommend more actions to be taken in order to reduce the use of avoidance coping strategies among victims of cyberbullying.

## 1. Introduction

According to a review by Aboujaoude et al. [1], several studies conducted in different countries, including the United States of America, China, Canada, Turkey, and the Netherlands, showed a prevalence of cybervictimization among adolescents. Although different measurements, definitions, and sample sizes were used, it is suggested that an estimated range between 20 and 40 percent of children and adolescents have been victims of aggressions through electronic means while 2 to 7 percent have been, at some time, victims of severe aggression. Rodríguez-Enríquez et al. [2] further suggested that adolescents aged between 12 and 15 years have the highest risk for cybervictimization, with a gradual decline in the risk at 16 years of age.

Burger and Bachmann [3] suggested that since cyberbullying is a subtype of bullying, its definition also needs to meet the three conditions of bullying, which are the intention to harm victims by the perpetrator, an imbalance in power between the perpetrator and victims, and repeated aggressive behaviors [3]. Although both cybervictimization and cyberbullying may imply a similarity, both carry different meanings. Burger and Bachmann [3] further explained that cyberbullying or bullying perpetration points to the act of performing any one or more of the three conditions of bullying actively, whereas cybervictimization or bullying victimization refers to being the target of such acts. Examples of cybervictimization provided by Wright and Wachs [4] encompass harassment, stalking, abuse, assault, hostility, happy slapping, outing, and flaming.

There have been different factors linked to cybervictimization. Demographic factors such as gender, ethnic groups, and age are relevant to cybervictimization. For example, in Morin et al. [5], an online survey among 28,583 US high school students was conducted and results found that white or Caucasian female upperclassmen (grades 11 and 12 vs. 9 and 10) and victims of school bullying or cyber perpetrators were more likely to be cybervictimized. Similarly, a survey by Strohmeier et al. [6] on 1018 Austria adolescents highlighted that females were more likely to be cybervictimized than males, and adolescents from first-generation immigrant families were also more likely to be cybervictimized when compared to non-immigrants. Moreover, in Mobin et al. [7], data collected from the Student Health Survey between 2010 and 2011 that included 5783 students from 109 elementary schools in Canada reported that aboriginal females from a higher grade who have smoked at least once in their life and were drinkers had a higher likelihood of being cybervictimized. Overall, findings from these studies suggest that females of an older age, and from ethnic minorities were more likely to be victims.

Besides demographic factors, studies also showed associations between personality traits and cybervictimization. Interestingly, some personality traits were also found to be positively associated with cybervictimization. In a study carried out by Rodríguez-Enríquez et al. [2] on 765 Spanish adolescents, victims scored higher in extraversion and emotional instability but lower in conscientiousness.

In addition to the factors related to demographics and personality, social development factors were also found to be important. For example, according to a study carried out by Charalampous et al. [8] on 868 early adolescents ranging from 10 to 15 years of age in public elementary and high schools located in Cyprus, the authoritarian parenting style had a positive association with cybervictimization. A survey conducted by Mobin et al. [7] among Canadian adolescents also reported that participants who had a poor relationship with parents and friends were more likely to experience cybervictimization.

Another factor that can also be evidently seen was the environment, such as school climate. Viau et al. [9] used a longitudinal survey to collect two waves of data from 2120 families in Canada. They reported that perception of a negative school climate was the only risk factor that had a consistent association with the experience of cyber and/or traditional victimization. Similarly, Azami and Taremian [10] surveyed 425 high school students from Iran also found that students who perceived poor school climates were more likely to be victims of this act.

Different negative consequences have been identified in association with cybervictimization. A cross-sectional design by Wang et al. [11] was used to survey 1324 Chinese middle and high school students. Results highlighted that cybervictimization was positively associated with non-suicidal self-injury. In Audrin and Blaya [12], their survey on 1019 children and young people between the ages of 9 and 17 in France showed the indication of a positive relation between cybervictimization to anxiety, self-esteem, impulsivity, and disruptive behavior. Rey et al. [13] also used a longitudinal design to collect two waves of data from a total of 1024 high school students aged 12 to 18 years in Spain, and they found that cybervictimization predicted somatic symptoms. In addition, a meta-analysis by Fisher et al. [14] of 55 reports that represented responses from 257,678 adolescents concluded that there were positive associations between cybervictimization and almost all of the internalized problems (suicidal ideation, depression, anxiety, self-esteem, and physical symptoms) as well as externalized problems (self-harm, substance use, and social problems).

Among the above-mentioned negative consequences, depression has been highlighted as a robust consequence by studies conducted in different countries. In China, a survey of 1006 Chinese adolescents from three junior middle schools located in the Guangdong province, South China, by Wang et al. [15] revealed a positive correlation between cybervictimization and depression. Similar results were reported by Xin et al. [16] in Guangdong province with 1006 Chinese adolescents, Niu et al. [17] in Central China with 755 Chinese adolescents from two junior high schools, and Li et al. [18] in Shanxi province with 793 Chinese adolescents.

Besides studies conducted in China, similar results pertaining to this association were also reported in other countries. In Spain, findings obtained from a survey on 1318 adolescents by Estévez et al. [19] from four secondary compulsory education centers reported that cybervictimization had a positive relationship with depression. Similar results were documented by Hellfeldt et al. [20], whose study was conducted among 1707 adolescents in Sweden, Williams et al. [21] on 233 high school students in the United States of America, Olenik-Shemesha et al. [22] on 242 adolescents in Israel, and Campbell et al. [23] on 3112 adolescents in Australia. In East London, a longitudinal design by Fahy et al. [24] collected two waves of data from 2480 teenagers. It was also found that cybervictims were significantly more likely to report depressive symptoms at a follow-up study one year after.

### 1.1. Mediators of Cybervictimization and Depression

Nonetheless, the relationship between cybervictimization and depression may be mediated by other factors. For example, Cohen et al. [25] surveyed 505 Israel adolescents and found that victims who experienced a high level of self-blame were prone to have depression and suicide ideations. This was similar to an online survey carried out by Prihadi et al. [26], where findings reported that both self-esteem and learned helplessness mediated the link between perceived cybervictimisation and perceived depression. Niu et al. [17] also revealed in their study on 755 Chinese adolescents that psychological security was a mediator between cybervictimization and depression, but a growth mindset could significantly alleviate the adverse effects of cyberbullying victimization on psychological security and on depression.

Coping strategies may also be a mediator for the effects of cybervictimization on depression. As suggested by Chan and Wong [27], the negative effects of cybervictimization were also caused by the coping approach employed by the victims. Hellfeldt et al. [20], however, emphasized that coping strategies could act as protective factors to mediate the relationship between cyberbullying and negative well-being.

The possible mediating role of coping on cybervictimization and depression is consistent with the prediction in the transactional model of stress and coping theory [28]. The Transactional Model of Stress and Coping suggests two processes of cognitive appraisal that mediate the influences of stressors on the consequences. The first cognitive appraisal is the primary appraisal, where individuals evaluate the significance of the event on an individual’s well-being, such as whether the event is threatening, challenging, or harmful. The secondary appraisal is initiated when individuals consider the resources and possible options to cope with the stressors [29]. The transactional model of stress and coping theory states that before an individual decides on the appropriate coping strategies to utilize, the individual will first appraise the level of stress brought on by the situation. According to Folkman [29], the appraisal is a continuous process of evaluation. During primary appraisal, the question, “Am I okay?” may be asked, while during secondary appraisal, the question “What can I do?” may be asked. Different coping strategies will be adopted in the secondary appraisal based on the results from the primary appraisal [29].

This then brings about the definition of coping, which is a continual change of thoughts and behaviors to manage specific external and internal demands that are appraised as stressful [29]. While a situation is appraised as an acceptable challenge, an individual, equipped with the resources and capabilities, is likely able to overcome it by using coping strategies that focus on problem-solving such as actively searching for solutions that may be helpful in facing it. Conversely, if an individual appraises the situation as a highly stressful one, coping strategies that are related to avoidance or delay in dealing with the tasks may be employed to temporarily reduce the stress [30].

A few coping strategies employed by the victims are elaborated in the ensuing studies. Heiman et al. [31] used focus group discussions to collect data from 26 students and, further, surveyed 232 students in Israel. Two categories of coping strategies, active and passive coping strategies, were identified from the thematic-narrative analysis. Active coping methods include seeking informative social support and counterattacking online in response to the attack, and passive coping methods include ignoring the cyberbullying and not approaching parents due to fear. Results from the survey indicated that when compared with those who were not cybervictims, less problem-focused coping strategies, and more emotion-focused coping strategies and avoidance coping strategies were used by victims. Some victims used the ignore or avoidance coping strategies, as they believed that the cyberbullying behavior would reduce on its own when these strategies were employed.

Some studies have highlighted the use of passive coping strategies, such as emotional or avoidance coping, among victims of cyberbullying. In Belgium, Vranjes et al. [32] surveyed 1715 students aged between 10 and 15 years old and found that adolescents who were cybervictimized more often suppressed or concealed their emotions. Mallmann et al. [33], in their survey on 273 students between 13 and 18 years old in Brazil, reported a significant association between cybervictimization and the escape-avoidance strategy. Chi et al. [34], meanwhile, reported that the majority of victims in Vietnam practiced the avoidance coping strategy by not paying attention and ignoring the situation of being cybervictimized as it was an online incident that was not serious and would eventually go away.

It was also observed from previous studies that victims rarely seek support from adults. Interestingly, the results from Chi et al. [34] on 215 students aged between 13 and 18 years old, and Ngo et al. [35] on 484 adolescents in Vietnam pointed out that more than half of the participants asked their friends for advice, but very few of them told their teachers. Heiman et al. [31] also reported that victims in Israel preferred to seek help from close friends instead of parents. The victims explained that their peers were more familiar with the cyber coping strategies, thus making their support more useful and applicable. The fear of being blamed for the occurrence of these incidents might have caused victims not to seek support from their parents. This was evident in Bradbury et al. [36], where they surveyed 329 seventh and eighth-grade students in the US and found that adolescents were usually reluctant to seek support from family or other adults. Similarly, Daneback et al. [37], who surveyed 451 adolescents in the Czech Republic, reported the same findings. Most victims did not want to seek social support as they thought this was an issue that could be managed by themselves. Some were unable to find someone whom they could trust and talk about this, while some had a fear of making the situation worse when the incident was made known.

On the contrary, some studies have also reported the use of active coping strategies. McLoughlin [38] surveyed 229 students aged between 12 and 17 years old in South Australia, which pointed out that the participants commonly practiced active coping, followed by distractions. This was similar to Ngo et al. [35], who did a survey on 484 adolescents in Vietnam and found that most adolescents told the cyberbullies to stop their actions. This was followed by informing their friends about this and logging out, leaving the platform, as well as stopping using the internet. On the other hand, data collected by Quintana-Orts et al. [39] in two waves from 979 adolescents in Spain showed that cybervictimization significantly predicted motivation for revenge. The motivation also significantly predicted cyber aggression, as at Time 1, it acted as a serial mediator between cybervictimization and cyberaggression at Time 2, four months after.

Some victims also used technology coping; Chi et al. [34] reported that blocking accounts was commonly employed among victims in Vietnam. Lee and Chun [40] also reported that some victims in South Korea used technology coping strategies such as changing their account names or profiles.

Besides the associations between cybervictimization and coping strategies, studies also revealed the associations between different coping strategies and their consequences. For example, in Hellfeldt et al. [20], the survey took place on 1707 youths aged between 10 and 13 years old in Sweden. For cyber victims, both individuals and in groups, higher levels of family and teachers’ support were negatively associated with depressive and anxiety symptoms but positively associated with higher levels of subjective well-being. Quintana-Orts et al. [39] reported that practicing forgiveness towards the perpetrators is an emotion-focused coping strategy intended to reduce negative emotions, thoughts, and behaviors after the cyberbullying experience. This corresponds with another study by Quintana-Orts and Rey [41], where a survey was conducted among 1650 students aged between 11 and 20 years old in Spain. The practice of forgiveness in coping with cyberbullying might reduce the possibility of future engagement in cyberbullying behaviors. In contrast, the act of forgiving might also be considered a negative coping strategy, as seen in a survey carried out by Rey et al. [13] on 251 cybervictims aged between 12 and 17 years old in Spain. Findings obtained reported that the act of forgiving was associated with depression. Thus, it is crucial to note that that the practice of forgiving perpetrators may have different outcomes on the victims.

Additionally, some studies also suggest that coping strategies are mediators for the effects of cybervictimization on certain psychological outcomes. For example, Trompeter et al. [42] found that coping self-efficacy mediated the effects of cybervictimization on depression and social anxiety. Jose and Vierling [43] proposed that rumination coping mediated the effects of cybervictimization on sleep adequacy, and Rey et al. [13] reported that self-blame and rumination coping strategies partially mediated the effects of cybervictimization on somatic symptoms.

### 1.2. Aims of Study

**Figure 1:**
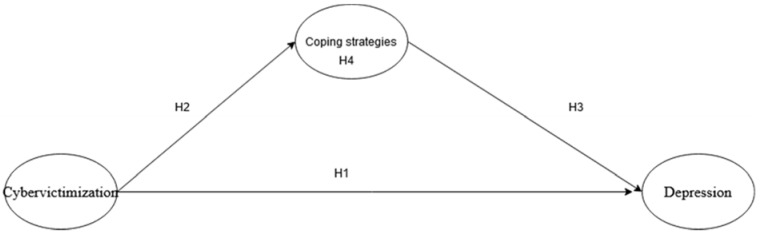
Conceptual Framework.

Studies have found associations between cybervictimization and depression [19,44], associations between cybervictimization and coping strategies [35,45], and an association between coping strategies and depression [13,20]. However, to our knowledge, only a few studies used the transactional model of stress and coping theory to examine coping strategies that are possible mediators of the effects of cybervictimization on depression. Hence, this model proposed by Lazrus and Folkman [28] was used to examine this research question. It is predicted that cybervictims would appraise the act of cyberbullying as a threat, leading to the adoption of different coping strategies based on their secondary appraisal which is related to the options to cope with cybervictimization. The adoption of these coping strategies would have different effects on their depression. In other words, the effects of cybervictimization on depression would be mediated by the coping strategies adopted. Active coping strategies, such as problem solving, seeking social support and technology coping, are predicted to be competitive mediators as they are negatively associated between cybervictimization and depression. On the other hand, the avoidance strategy, which falls under the passive coping strategies is predicted to be a complementary mediator as it is positively associated with depression. The competitive mediator is when the mediated effect and direct effect both exist and point at different directions, while the complementary mediator is when the mediated effect and direct effect both exist and point in the same direction [46]. Below are the research hypotheses and conceptual framework (see Figure 1).

**Hypothesis** **1** **(H1).** *Cybervictimization is positively associated with depression*.

**Hypothesis** **2** **(H2).** *Cybervictimization is positively associated with different coping strategies (active and avoidance coping strategies)*.

**Hypothesis** **3** **(H3).** *Coping strategies are associated with depression*.

**Hypothesis** **3a** **(H3a).** *Active coping strategies (Problem-solving, social support, and technology coping) are negatively associated with depression*.

**Hypothesis** **3b** **(H3b).** *Avoidance coping strategy is positively associated with depression*.

**Hypothesis** **4** **(H4).** *Coping strategies are statistical mediators on the effects of cybervictimization on depression*.

**Hypothesis** **4a** **(H4a).** *Active coping strategies are competitive mediators on the effects of cybervictimization on depression*.

**Hypothesis** **4b** **(H4b).** *Avoidance coping strategy is a complementary mediator on the effects of cybervictimization on depression*.

## 2. Materials and Methods

### 2.1. Participants

A total of 436 secondary school students aged between 13 and 15 participated in the study. However, after screening out 41 questionnaires with response bias (similar responses to all items) and 8 questionnaires with over 5% missing data [47], the final valid sample was 387 (age M = 14.35, SD = 0.86). In terms of ethnicity, 45.1% were Chinese, 33.6% were Malays, 20.3% were Indians, and 1% were from other ethnic groups.

### 2.2. Measurements

The measurements without Malay and Chinese versions were translated from English into two languages using a back-translation method [48]. After translating to these languages, the questionnaire was then translated into English by a researcher who was blinded to the original questionnaire. Both original and translated versions in English were compared. Items with different meanings in both versions were revised until a consensus was reached on the finalized version. The internal consistencies of both versions of translated measurements are all above 0.70.

Below are the sections contained in the questionnaire.

Demographic information. Participants were required to fill in their personal background information, including age, gender, ethnicity, and education level.

Cybervictimization. The cybervictimization subscale of the European Cyberbullying Intervention Project Questionnaire [49,50] was used to assess cybervictimization. A total of 11 items were rated on a 5-point Likert scale, ranging from “0 = never”, “1 = once or twice”, “2 = once or twice a month”, “3 = once a week”, and “4 = more than once a week”. Sample items include “someone hacked into my account and pretended to be me” and “someone spread rumors about me through online voice messages”. The reliability of the measurement is reported at Cronbach’s alpha 0.86 [51]. A higher mean score indicated more cybervictimization experience [52].

Coping Strategies. According to a finding reported by McLoughlin [38], Brief COPE is an adequate measure of coping with cyberbullying among adolescents. The Brief COPE consists of 28 items [53]. There are 4 factors for the scale: social support (8 items), problem-solving (4 items), avoidance (10 items), and positive thinking (6 items) [54]. Sample items of each factor are “I’ve been getting comfort and understanding from someone” and “I’ve been getting help and advice from other people” (social support), “I’ve been taking action to try to make the situation better”, and “I’ve been thinking hard about what steps to take“(problem-solving), “I’ve been using alcohol or other drugs to help me get through it”, and “I’ve been refusing to believe that it has happened” (avoidance), and “I’ve been making jokes about it”, and “I’ve been learning to live with it“ (positive thinking). The Cronbach alpha of social support was reported at 0.82, 0.74 for problem-solving, 0.64 for avoidance, and 0.71 for positive thinking [54]. The Malay version was adapted from Yusoff [55], while the Chinese version was adapted from Chiu [56]. Six items of technological coping by Machackova et al. [57] were also added. Participants were instructed to respond by selecting the most appropriate response based on a 4-point Likert Scale from 0 to 3 on how they cope with cyberbullying. Sample items include “I reported this to the administrator” and “deleted my profile on the web pages where this happened”. Participants reported their reactions when they faced a stressful event on this scale, namely “0 = I haven’t been doing this at all”, “1 = I’ve been doing this for a little bit”, “2 = I’ve been doing this a medium amount”, or “3 = I’ve been doing this a lot”. A higher mean score indicated a higher tendency to apply certain coping strategies. The reliability of technological coping was reported at Cronbach’s alpha 0.87 [58].

Depression. The Short Mood and Feelings Questionnaire included 13 items that assessed the affective and cognitive symptoms of depression [59]. The items were rated on a 3-point Likert scale, namely “0 = not true”, “1 = sometimes true”, or “2 = true” over the past two weeks, with a maximum total score of 26. A sample item was “I felt miserable or unhappy” and “I found it hard to think properly or concentrate”. A higher mean score indicated a higher level of depression. The reliability of the scale was reported at Cronbach’s alpha 0.85 [59]. The Chinese version translated by He and Shi was provided by Dr. Brian Small through personal email communication.

Control variables. Among the 5 demographic variables, the results of Spearman correlation showed that only languages (Malay vs. Chinese) and ethnicity (Chinese vs. Non-Chinese) were significantly correlated with depression, r (385) = −0.15, *p* = 0.004 for language and r (385) = −0.21, *p* < 0.001 for ethnicity. Accordingly, these two variables were used as control variables.

### 2.3. Procedure

After getting approval from the university’s Scientific and Ethical Committee, Ministry of Education, and State Education Departments, school principals from schools located in the northern zone, central zone, and southern zone in Malaysia were contacted from a list to obtain permission for the questionnaire distribution. Four secondary schools agreed to assist the distribution of questionnaires to their students.

After obtaining approval from the school principals, the questionnaires were prepared and sent to contact teachers at the respective schools. Data collection was carried out from August to December 2021. Prior to the distribution, a briefing was given to the contact teachers to explain the purpose of this study as well as the inclusive and exclusive criteria for students to participate in the survey. The purposive sampling method was used where only participants aged between 13 and 15 years of age were selected. As they were below 18 years old, contact teachers were reminded to only recruit students who returned the signed parental consent form for the survey. Teachers were also reminded to read and explain the content of the informed consent form on the first page to students before they answered the questionnaire. The informed consent form included information such as the aims of the survey, their right to withdraw from the survey at any time, assurance of confidentiality of responses. Students who did not give their consent, even though they had parental consent, were not recruited for this survey.

Besides the printed questionnaires, students were also given an option to answer the questionnaire online in a Google form. The link to this form was sent to the contact teachers and shared with students who met similar inclusive and exclusive criteria. Participants took about 15 to 20 min to complete the questionnaire. Overall, 72.1% of valid data were collected from printed questionnaires and 27.9% from the online version. 62.8% of participants answered the Malay version, and 37.2% answered the Chinese version of the questionnaire. After completing the questionnaire, a bookmark was given as a token of appreciation for their participation.

### 2.4. Data Analyses

The descriptive results were analyzed by the SPSS program, and the Partial Least Squares Structural Equation Modeling was analyzed by the SmartPLS program. The SPSS program with Mardia macro was used to examine the normality of data through an examination of the multivariate skewness and kurtosis [60]. Besides, Spearman correlation was used to examine the relationships among demographic variables and depression for the selection of control variables. Then, the SmartPLS program was used to examine the measurement model, and a structural model was conducted next to examine the relationships among the variables [61]. The decision to use the Partial Least Squares Structural Equation Modeling was based on the software itself. It is a non-parametric analysis software and could meet the aim of testing a theoretical framework from a predictive perspective [62,63,64].

## 3. Results

### 3.1. Data Cleaning

The Mardia macro was run using the SPSS program (Version 21) to examine the normality of data [60]. The results indicated rejection of the null hypothesis as the data was not multivariate normal, Mardia’s multivariate skewness (β = 9.23, *p* < 0.001) and Mardia’s multivariate kurtosis (β = 59.16, *p* < 0.001). Based on the suggestions from Hair et al. [64] and Ramayah et al. [63], the SmartPLs program, a non-parametric analysis software, was used to examine the measurement and structural model of the study. Bootstrapping method with 5000 resamples was also used to test the significance of the path coefficients. Additionally, the mean replacement was used to handle questionnaires with less than 5 percent of missing data [47].

### 3.2. Measurement Model

#### 3.2.1. Construct Reliability and Discriminant Validity

Composite reliability was used to examine the scales’ internal consistency, as it was more appropriate for the Partial Least Square Structural Equation Model since the formula considered different outer loadings on the construct [47]. As shown in Table 1, after removing 3 items from the avoidance scale, all the scales’ composite reliability ranged from 0.87 to 0.92, where it exceeded the recommended value of 0.7 [47]. The 3 items were removed to improve the Average Variance Extracted to over 0.5 thresholds, as suggested by Wong [65]. Correspondingly, the findings suggested that the latent constructs of all scales were at an acceptable level. However, the heterotrait-monotrait ratio of positive thinking and problem solving was 0.92, higher than the critical values of 0.90 [66]. Following the suggestion of Henseler et al. [66], these constructs were merged to be positive thinking as it could be a method of problem-solving. After combining both constructs, all measurements’ heterotrait-monotrait ratios are below the critical values of 0.90, indicating that the discriminant validities of all measurements were acceptable (Table 1).

#### 3.2.2. Coefficient of Determination, Effect Size, and Collinearity Statistics of Measurements

As shown in Table 2, a large effect size was also found on depression, r^2^ = 0.38. However, only a middle to large effect was found in the association between avoidance and depression, f^2^ = 0.20. Besides, there was no collinearity issue as the variance inflation factor of all predictors was also below 5 [67].

### 3.3. Structural Model

After controlling the gender variable, the one-tailed test, bootstrapping results with 5000 samples revealed that cybervictimization was positively associated with all types of coping strategies, *ps* < 0.001. However, only avoidance coping was positively associated with depression, and social support was negatively associated with depression, *ps* < 0.05. Besides, cybervictimization was not significantly associated with depression, *p* = 0.131 (Table 3).

#### Mediating Effects

The decision tree proposed by Zhao et al. [46] was used to examine the mediating effects of coping strategies. As indicated in Table 4, the bootstrapping results with 5000 samples showed significant specific indirect effects of avoidance on the effects of cybervictimization on depression, *p* < 0.001. Besides, the results also showed significant specific indirect effects of social support on the effects of cybervictimization on depression, *p* = 0.047. As the direct effect of victimization on depression was not significant, *p* = 0.13, these results indicated an indirect mediating effect of avoidance and social support on the effects of victimization on depression.

## 4. Discussion

Researchers have reported associations between cybervictimization and depression [19,22,44], cybervictimization and coping [35,38,45], and coping and depression [20,41]. Findings also suggested a mechanism that linked the three variables. Nevertheless, to our knowledge, only a few studies were conducted to examine this issue by using the transactional model of stress and coping theory [28] as a framework. Based on this theory, it was indicated that coping strategies could be a mediator on the effects of cybervictimization on depression.

Firstly, similar to other studies, Hellfeldt et al. [20] and Quintana-Orts and Rey [41], the results also supported the different associations between coping strategies and depression. However, the results showed that not all coping strategies are associated with depression. Only the avoidant coping strategy was positively associated with depression, while the social support strategy was negatively associated with depression. The negative association between avoidance coping and depression showed a robust result, as reported in different studies conducted on different populations, including those in same-sex relationships [68,69], war veterans [70], pregnant women [71], undergraduates [72], children and adolescents with cancer [73]. Besides, the positive association between social support and depression was also reported on different populations, including hospital nurses [74], senior citizens [75], and young adolescents [20].

Results from this study revealed that the two active coping strategies, problem-solving and technological coping, were not associated with depression. These could be related to the effectiveness of the two active coping strategies mentioned above. In our interviews with 18 victims of cyberbullying [76], a problem-solving strategy mentioned by a victim was to fight back, such as “throwing racist comments back to them”. Besides, technological coping may not be effective for the bullying that had a combination of school bullying and cyberbullying [77].

Secondly, the results also highlighted significant associations between cybervictimization and the four coping strategies. These results are consistent with studies that reported significant associations between various coping strategies adopted by victims of cyberbullying. These coping strategies include social support [45], active coping [38], confronting [35], technological coping [34], and avoidance [32].

Thirdly, the results did not support the positive association between cyber victimization and depression. These results, however, are different from studies that reported significant associations between these two variables [19,22,44]. A possible reason behind this is an indirect rather than a direct effect of cybervictimization on depression, where the association between these two variables was mediated or partially mediated through other variables. For instance, McLoughlin et al. [78] found that social connectedness partially mediated the relationship between frequent cybervictimization and depression, and Chu et al. [79] reported that hopelessness was a partial mediator in this relationship. Besides, the non-significant association may also be relevant to the different measurements used. For example, Estévez et al. [19] reported a significant association between cybervictimization and depression by using the Cybervictimization Scale and the Depression Scale of the Center of Epidemiological Studies, but Boer et al. (2021) did not find such association by using the Multidimensional Online Peer Victimization Scale and Depressive Mood List.

The results of mediating analysis reported that not all coping strategies were mediators of the effects of cybervictimization on depression. The only significant mediating effect was found in the avoidance coping strategy. Thus, it can be highlighted that victims would use different strategies to cope with cyberbullying, but only those who frequently used the avoidance coping strategy would lead to depression. These findings explained the non-significant association between cybervictimization and depression that was found in this study. This is also explained in the transactional model of stress and coping theory by Lazarus and Folkman [28], where the use of different coping strategies to overcome various threats may lead to different outcomes.

### 4.1. Implications

Implications from the theoretical and practical aspects include the support on the application of the model for this study and proposed actions to help the victims. This study’s findings suggested that the transactional model of stress and coping theory [28] may provide a framework to understand the mechanism pertaining to the association between cybervictimization and negative psychological outcomes. It is also important to note that not all coping strategies mediate the effects of cybervictimization and depression. As previous studies categorized the coping measurements and coping strategies in different ways, it may be pertinent that a coping measurement that covers all coping strategies for victims of cyberbullying be developed for the purpose of uniformity.

From the practical perspective, the findings suggest more studies should be performed to explore the impact of an intervention to discourage the use of avoidance coping strategies among victims of cyberbullying, since it has been reported as a frequently used strategy by victims of cyberbullying in studies carried out in different countries, such as Belgium, Brazil, and Vietnam [32,33,34]. The non-significant mediating effects of active coping strategies may indicate the ineffectiveness of these strategies adopted by victims to cope with cyberbullying. Additionally, studies conducted on adolescents from different countries, i.e., the United States of America, Germany, Vietnam, and the Czech Republic, revealed similar results where victims preferred to seek advice and help from their peers than adults [34,36,37]. It is strongly recommended that parents and teachers be more involved in helping adolescents deal with this issue effectively. Training sessions may be provided to guide parents and teachers to be more mindful of the symptoms shown by adolescents when they are cyberbullied and provide a safe environment for them to seek help.

### 4.2. Limitations

The interpretation of the findings should be treated with caution. As purposive sampling was used to examine the relationships among variables, the findings may not be generalizable to the whole population. Recruitment of different samples is needed to examine the robustness of the findings. The cross-sectional design used in this study to explain the cause-effect needs to be looked at cautiously as findings from this study are based on a statistical model and may not be able to meet the conditions of a cause-effect explanation, such as the time-order relationship and the elimination of alternative causal explanation [80]. Besides, there are other alternative models that may explain the associations among the variables, such as depression or adoption of coping strategies by the victims that make them more likely to experience cybervictimization. Future studies may consider a different theoretical framework and design to examine this possibility. In addition, about 10 percent of distributed questionnaires with response bias and the use of mean value replacement may also limit the variability of the data and cause a reduction in the possibility of finding meaningful relationships among the variables in this study. Moreover, since there are different measurements for cyberbullying behaviors [81], this may limit the generalization and robustness of the current findings.

## 5. Conclusions

In conclusion, the findings show the associations between the adoption of various strategies by victims to cope with cyberbullying and depression. However, the adoption of avoidance coping strategies and social support were more likely to be associated with depression.

## Figures and Tables

**Table 1:** Composite reliability, average variance extracted (AVE), and discriminant validity of measurements.

	Total Items	Mean	SD	Composite Reliability	AVE	1	2	3	4	5
1. Avoidance	7 *	0.86	0.70	0.87	0.50					
2. Problem solving	10	1.14	0.84	0.92	0.52	0.81				
3. Technological	6	0.75	0.77	0.90	0.61	0.65	0.61			
4. Social support	8	1.05	0.79	0.91	0.55	0.73	0.87	0.59		
5. Depression	12	0.61	0.50	0.92	0.50	0.69	0.46	0.42	0.39	
6. Victimization	11	0.55	0.69	0.92	0.51	0.62	0.42	0.56	0.40	0.40

Note: * three items removed.

**Table 2:** Coefficient of determination (r^2^), effect size (f^2^), and collinearity statistics (VIF) of Measurements.

Exogenous	Endogenous	r^2^	f^2^	VIF
Avoidance		0.31		
	Cybervictimization		0.45	1.00
Problem-solving		0.15		
	Cybervictimization		0.17	1.00
Technological		0.25		
	Cybervictimization		0.34	1.00
Social support		0.15		
	Cybervictimization		0.18	1.00
Depression		0.38		
	Avoidance		0.20	2.48
	Problem-solving		0.01	3.22
	Technological		0.01	1.72
	Social support		0.01	2.78
	Cybervictimization		0.01	1.60

**Table 3:** Results of direct effect analyses.

	Hypothesis	Std. Beta	SE	T-Values	*p*-Values	95% Percentile Confidence Interval
**Cybervictimization → Coping**						
Cybervictimization → Avoidance	H1	0.58	0.04	15.87	<0.001	[0.519, 0.639]
Cybervictimization → Problem-solving	H1	0.39	0.04	9.29	<0.001	[0.324, 0.460]
Cybervictimization → Technological	H1	0.52	0.04	12.25	<0.001	[0.456, 0.596]
Cybervictimization → Social support	H1	0.36	0.04	8.06	<0.001	[0.288, 0.435]
**Coping → Depression**						
Avoidance → Depression	H2	0.55	0.07	8.08	<0.001	[0.435, 0.663]
Problem solving → Depression	H2	0.07	0.08	0.83	0.202	[−0.064, 0.206]
Technological → Depression	H2	0.05	0.06	0.87	0.192	[−0.049, 0.158]
Social support → Depression	H2	−0.12	0.07	1.68	0.047	[−0.242, 0.001]
Cybervictimization → Depression	H3	0.07	0.06	1.12	0.131	[−0.035, 0.167]
**Control Variables**						
Languages → Depression		−0.04	0.04	0.95	0.171	[−0.113, 0.030]
Languages → Avoidance		0.10	0.05	2.11	0.017	[0.021, 0.173]
Languages → Problem-solving		0.10	0.05	1.77	0.038	[0.005, 0.186]
Languages → Technology		0.08	0.05	1.55	0.060	[−0.005, 0.155]
Languages → Social support		−0.03	0.06	0.54	0.295	[−0.119, 0.062]
Ethnicity → Depression		−0.06	0.05	1.31	0.094	[−0.133, 0.016]
Ethnicity → Avoidance		−0.21	0.04	5.11	0.000	[−0.275, 0.140]
Ethnicity → Problem-solving		−0.28	0.04	6.94	0.000	[−0.345, −0.212]
Ethnicity → Technology		−0.09	0.04	2.28	0.011	[−0.150, −0.024]
Ethnicity → Social support		−0.28	0.04	6.74	0.000	[−0.353, −0.215]

**Table 4:** Results of mediating analyses.

	Std. Beta	SE	T-Values	*p*-Values	95% Percentile Confidence Interval
Cybervictimization → Technological → Depression	0.03	0.03	0.84	0.199	[−0.026, 0.085]
Cybervictimization → Avoidance → Depression	0.32	0.04	7.44	<0.001	[0.251, 0.391]
Cybervictimization → Problem solving → Depression	0.03	0.03	0.81	0.210	[−0.024, 0.084]
Cybervictimization → Social support → Depression	−0.04	0.03	1.67	0.047	[−0.087, 0.000]

## Data Availability

The data presented in this study are available on request from the corresponding author. The data are not publicly available due to privacy agreements.

## References

[1] Aboujaoude E., Savage M.W., Starcevic V., Salame W.O. (2015). Cyberbullying: Review of an Old Problem Gone Viral. J. Adolesc. Health.

[2] Rodríguez-Enríquez M., Bennasar-Veny M., Leiva A., Garaigordobil M., Yañez A.M. (2019). Cybervictimization among Secondary Students: Social Networking Time, Personality Traits and Parental Education. BMC Public Health.

[3] Burger C., Bachmann L. (2021). Perpetration and Victimization in Offline and Cyber Contexts: A Variable-and Person-Oriented Examination of Associations and Differences Regarding Domain-Specific Self-Esteem and School Adjustment. Int. J. Environ. Res. Public Health.

[4] Wright M.F., Wachs S. (2020). Adolescents’ Cyber Victimization: The Influence of Technologies, Gender, and Gender Stereotype Traits. Int. J. Environ. Res. Public Health.

[5] Morin H.K., Bradshaw C.P., Kush J.M. (2018). Adjustment Outcomes of Victims of Cyberbullying: The Role of Personal and Contextual Factors. J. Sch. Psychol..

[6] Strohmeier D., Gradinger P., Yanagida T. (2021). The Role of Intrapersonal-, Interpersonal-, Family-, and School-Level Variables in Predicting Bias-Based Cybervictimization. J. Early Adolesc..

[7] Mobin A., Feng C.X., Neudorf C. (2017). Cybervictimization among Preadolescents in a Community-Based Sample in Canada: Prevalence and Predictors. Can. J. Public Health.

[8] Charalampous K., Demetriou C., Tricha L., Ioannou M., Georgiou S., Nikiforou M., Stavrinides P. (2018). The Effect of Parental Style on Bullying and Cyber Bullying Behaviors and the Mediating Role of Peer Attachment Relationships: A Longitudinal Study. J. Adolesc..

[9] Viau S.-J., Denault A.-S., Dionne G., Brendgen M., Geoffroy M.-C., Côté S., Larose S., Vitaro F., Tremblay R.E., Boivin M. (2020). Joint Trajectories of Peer Cyber and Traditional Victimization in Adolescence: A Look at Risk Factors. J. Early Adolesc..

[10] Azami M.S., Taremian F. (2021). Risk Factors Associated with Cyberbullying, Cybervictimization, and Cyberbullying-Victimization in Iran’s High School Students. Iran. J. Psychiatry.

[11] Wang Y., Chen A., Ni H. (2020). The Relationship between Cybervictimization and Non-Suicidal Self-Injury in Chinese Adolescents: A Moderated-Mediation Model. Front. Psychol..

[12] Audrin C., Blaya C. (2020). Psychological Well-Being in a Connected World: The Impact of Cybervictimization in Children’s and Young People’s Life in France. Front. Psychol..

[13] Rey L., Neto F., Extremera N. (2020). Cyberbullying Victimization and Somatic Complaints: A Prospective Examination of Cognitive Emotion Regulation Strategies as Mediators. Int. J. Clin. Health Psychol..

[14] Fisher B.W., Gardella J.H., Teurbe-Tolon A.R. (2016). Peer Cybervictimization among Adolescents and the Associated Internalizing and Externalizing Problems: A Meta-Analysis. J. Youth Adolesc..

[15] Wang Z., Xie Q., Xin M., Wei C., Yu C., Zhen S., Liu S., Wang J., Zhang W. (2020). Cybervictimization, Depression, and Adolescent Internet Addiction: The Moderating Effect of Prosocial Peer Affiliation. Front. Psychol..

[16] Xin M., Chen P., Liang Q., Yu C., Zhen S., Zhang W. (2021). Cybervictimization and Adolescent Internet Addiction: A Moderated Mediation Model. Int. J. Environ. Res. Public Health.

[17] Niu G., He J., Lin S., Sun X., Longobardi C. (2020). Cyberbullying Victimization and Adolescent Depression: The Mediating Role of Psychological Security and the Moderating Role of Growth Mindset. Int. J. Environ. Res. Public Health.

[18] Li Y., Li D., Li X., Zhou Y., Sun W., Wang Y., Li J. (2018). Cyber Victimization and Adolescent Depression: The Mediating Role of Psychological Insecurity and the Moderating Role of Perceived Social Support. Child. Youth Serv. Rev..

[19] Estévez J.F., Cañas E., Estévez E. (2020). The Impact of Cybervictimization on Psychological Adjustment in Adolescence: Analyzing the Role of Emotional Intelligence. Int. J. Environ. Res. Public Health.

[20] Hellfeldt K., López-Romero L., Andershed H. (2020). Cyberbullying and Psychological Well-Being in Young Adolescence: The Potential Protective Mediation Effects of Social Support from Family, Friends, and Teachers. Int. J. Environ. Res. Public Health.

[21] Williams S.G., Langhinrichsen-Rohling J., Wornell C., Finnegan H. (2017). Adolescents Transitioning to High School: Sex Differences in Bullying Victimization Associated with Depressive Symptoms, Suicide Ideation, and Suicide Attempts. J. Sch. Nurs..

[22] Olenik-Shemesh D., Heiman T., Eden S. (2012). Cyberbullying Victimisation in Adolescence: Relationships with Loneliness and Depressive Mood. Emot. Behav. Diffic..

[23] Campbell M., Spears B., Slee P., Butler D., Kift S. (2012). Victims’ Perceptions of Traditional and Cyberbullying, and the Psychosocial Correlates of Their Victimisation. Emot. Behav. Diffic..

[24] Fahy A.E., Stansfeld S.A., Smuk M., Smith N.R., Cummins S., Clark C. (2016). Longitudinal Associations between Cyberbullying Involvement and Adolescent Mental Health. J. Adolesc. Health.

[25] Cohen O.B.S., Shahar G., Brunstein Klomek A. (2020). Peer Victimization, Coping Strategies, Depression, and Suicidal Ideation among Young Adolescents. Crisis J. Crisis Interv. Suicide Prev..

[26] Prihadi K., Hui Y.L., Chua M., Chang C.K. (2019). Cyber-Victimization and Perceived Depression: Serial Mediation of Self-Esteem and Learned-Helplessness. Int. J. Eval. Res. Educ..

[27] Chan H.C.O., Wong D.S. (2017). Coping with Cyberbullying Victimization: An Exploratory Study of Chinese Adolescents in Hong Kong. Int. J. Law Crime Justice.

[28] Lazarus R.S., Folkman S. (1984). Stress, Appraisal, and Coping.

[29] Folkman S., Gellman M.D., Turner J.R. (2013). Stress: Appraisal and Coping. Encyclopedia of Behavioral Medicine.

[30] Sirois F.M., Kitner R. (2015). Less Adaptive or More Maladaptive? A Meta-Analytic Investigation of Procrastination and Coping. Eur. J. Personal..

[31] Heiman T., Olenik-Shemesh D., Frank G. (2019). Patterns of Coping with Cyberbullying: Emotional, Behavioral, and Strategic Coping Reactions among Middle School Students. Violence Vict..

[32] Vranjes I., Erreygers S., Vandebosch H., Baillien E., De Witte H. (2018). Patterns of Cybervictimization and Emotion Regulation in Adolescents and Adults. Aggress. Behav..

[33] Mallmann C.L., de Macedo Lisboa C.S., Zanatta Calza T. (2018). Cyberbullying and Coping Strategies in Adolescents from Southern Brazil. Acta Colomb. Psicol..

[34] Chi P.T.L., Lan V.T.H., Ngan N.H., Linh N.T. (2020). Online Time, Experience of Cyber Bullying and Practices to Cope with It among High School Students in Hanoi. Health Psychol. Open.

[35] Ngo A.T., Tran A.Q., Tran B.X., Nguyen L.H., Hoang M.T., Nguyen T.H.T., Doan L.P., Vu G.T., Nguyen T.H., Do H.T. (2021). Cyberbullying among School Adolescents in an Urban Setting of a Developing Country: Experience, Coping Strategies, and Mediating Effects of Different Support on Psychological Well-Being. Front. Psychol..

[36] Bradbury S.L., Dubow E.F., Domoff S.E. (2018). How Do Adolescents Learn Cyber-Victimization Coping Skills? An Examination of Parent and Peer Coping Socialization. J. Youth Adolesc..

[37] Daneback K., Bjereld Y., Macháčková H., Ševčíková A., Dědková L. (2018). Bullied Online but Not Telling Anyone: What Are the Reasons for Not Disclosing Cybervictimization?. Studia Paedagog..

[38] McLoughlin L.T. (2019). Understanding and Measuring Coping with Cyberbullying in Adolescents: Exploratory Factor Analysis of the Brief Coping Orientation to Problems Experienced Inventory. Curr. Psychol..

[39] Quintana-Orts C., Rey L., Chamizo-Nieto M.T., Worthington E.L. (2020). A Serial Mediation Model of the Relationship between Cybervictimization and Cyberaggression: The Role of Stress and Unforgiveness Motivations. Int. J. Environ. Res. Public Health.

[40] Lee S., Chun J. (2020). Conceptualizing the Impacts of Cyberbullying Victimization among Korean Male Adolescents. Child. Youth Serv. Rev..

[41] Quintana-Orts C., Rey L. (2018). Forgiveness and Cyberbullying in Adolescence: Does Willingness to Forgive Help Minimize the Risk of Becoming a Cyberbully?. Comput. Hum. Behav..

[42] Trompeter N., Bussey K., Fitzpatrick S. (2018). Cyber Victimization and Internalizing Difficulties: The Mediating Roles of Coping Self-Efficacy and Emotion Dysregulation. J. Abnorm. Child Psychol..

[43] Jose P.E., Vierling A. (2018). Cybervictimisation of Adolescents Predicts Higher Rumination, Which in Turn, Predicts Worse Sleep over Time. J. Adolesc..

[44] Gao L., Liu J., Yang J., Wang X. (2021). Longitudinal Relationships among Cybervictimization, Peer Pressure, and Adolescents’ Depressive Symptoms. J. Affect. Disord..

[45] Holfeld B., Baitz R. (2020). The Mediating and Moderating Effects of Social Support and School Climate on the Association between Cyber Victimization and Internalizing Symptoms. J. Youth Adolesc..

[46] Zhao X., Lynch J.G., Chen Q. (2010). Reconsidering Baron and Kenny: Myths and Truths about Mediation Analysis. J. Consum. Res..

[47] Hair J.F., Hult G.T.M., Ringle C., Sarstedt M. (2016). A Primer on Partial Least Squares Structural Equation Modeling (PLS-SEM).

[48] World Health Organization Process of Translation and Adaptation of Instruments. https://www.who.int/substance_abuse/research_tools/translation/en/.

[49] Del Rey R., Casas J.A., Ortega-Ruiz R., Schultze-Krumbholz A., Scheithauer H., Smith P., Thompson F., Barkoukis V., Tsorbatzoudis H., Brighi A. (2015). Structural Validation and Cross-Cultural Robustness of the European Cyberbullying Intervention Project Questionnaire. Comput. Hum. Behav..

[50] Brighi A., Ortega R., Pyzalski J., Scheithauer H., Smith P.K., Tsormpatzoudis H., Barkoukis V., Del Rey R., Guarini A., Plichta P. (2012). European Cyberbullying Intervention Project Questionnaire (ECIPQ). Unpubl. Quest..

[51] Martínez-Monteagudo M.C., Delgado B., Díaz-Herrero Á., García-Fernández J.M. (2020). Relationship between Suicidal Thinking, Anxiety, Depression and Stress in University Students Who Are Victims of Cyberbullying. Psychiatry Res..

[52] Erreygers S., Vandebosch H., Vranjes I., Baillien E., De Witte H. (2018). Positive or Negative Spirals of Online Behavior? Exploring Reciprocal Associations between Being the Actor and the Recipient of Prosocial and Antisocial Behavior Online. New Media Soc..

[53] Carver C.S. (1997). You Want to Measure Coping but Your Protocol’ Too Long: Consider the Brief Cope. Int. J. Behav. Med..

[54] Baumstarck K., Alessandrini M., Hamidou Z., Auquier P., Leroy T., Boyer L. (2017). Assessment of Coping: A New French Four-Factor Structure of the Brief COPE Inventory. Health Qual. Life Outcomes.

[55] Yusoff M.S.B. (2011). The Validity of the Malay Brief COPE in Identifying Coping Strategies among Adolescents in Secondary School. Int. Med. J..

[56] Chiu I.T. (2018). The Effect of Problem Behaviors of Children with Autism Spectrum Disorder and Coping Strategies of Mothers on Their Depression and Quality of Life. Master’s Thesis.

[57] Machackova H., Cerna A., Sevcikova A., Dedkova L., Daneback K. (2013). Effectiveness of Coping Strategies for Victims of Cyberbullying. Cyberpsychology.

[58] Horwitz A.G., Hill R.M., King C.A. (2011). Specific Coping Behaviors in Relation to Adolescent Depression and Suicidal Ideation. J. Adolesc..

[59] Messer S.C., Angold A., Costello E.J., Loeber R., Van Kammen W., Stouthamer-Loeber M. (1995). Development of a Short Questionnaire for Use in Epidemiological Studies of Depression in Children and Adolescents: Factor Composition and Structure across Development. Int. J. Methods Psychiatr. Res..

[60] Cain M.K., Zhang Z., Yuan K.-H. (2017). Univariate and Multivariate Skewness and Kurtosis for Measuring Nonnormality: Prevalence, Influence and Estimation. Behav. Res. Methods.

[61] Wong K.K.-K. (2013). Partial Least Squares Structural Equation Modeling (PLS-SEM) Techniques Using SmartPLS. Mark. Bull..

[62] Willaby H.W., Costa D.S., Burns B.D., MacCann C., Roberts R.D. (2015). Testing Complex Models with Small Sample Sizes: A Historical Overview and Empirical Demonstration of What Partial Least Squares (PLS) Can Offer Differential Psychology. Personal. Individ. Differ..

[63] Ramayah T., Yeap J.A., Ahmad N.H., Halim H.A., Rahman S.A. (2017). Testing a Confirmatory Model of Facebook Usage in Smartpls Using Consistent PLS. Int. J. Bus. Innov..

[64] Hair J.F., Risher J.J., Sarstedt M., Ringle C.M. (2019). When to Use and How to Report the Results of PLS-SEM. Eur. Bus. Rev..

[65] Wong K.K. (2016). Mediation Analysis, Categorical Moderation Analysis, and Higher-Order Constructs Modeling in Partial Least Squares Structural Equation Modeling (PLS-SEM): A B2B Example Using SmartPLS. Mark. Bull..

[66] Henseler J., Ringle C.M., Sarstedt M. (2015). A New Criterion for Assessing Discriminant Validity in Variance-Based Structural Equation Modeling. J. Acad. Mark. Sci..

[67] Hadi N.U., Abdullah N., Sentosa I. (2016). Making Sense of Mediating Analysis: A Marketing Perspective. Rev. Integr. Bus. Econ. Res..

[68] Bergfeld J.R., Chiu E.Y. (2017). Mediators in the Relationship between Minority Stress and Depression among Young Same-Sex Attracted Women. Prof. Psychol. Res. Pract..

[69] Cherenack E.M., Sikkema K.J., Watt M.H., Hansen N.B., Wilson P.A. (2018). Avoidant Coping Mediates the Relationship between Self-Efficacy for HIV Disclosure and Depression Symptoms among Men Who Have Sex with Men Newly Diagnosed with HIV. AIDS Behav..

[70] Bartone P.T., Homish G.G. (2020). Influence of Hardiness, Avoidance Coping, and Combat Exposure on Depression in Returning War Veterans: A Moderated-Mediation Study. J. Affect. Disord..

[71] Giurgescu C., Zenk S.N., Templin T.N., Engeland C.G., Dancy B.L., Park C.G., Kavanaugh K., Dieber W., Misra D.P. (2015). The Impact of Neighborhood Environment, Social Support, and Avoidance Coping on Depressive Symptoms of Pregnant African-American Women. Women’s Health Issues.

[72] Grant D.M., Wingate L.R., Rasmussen K.A., Davidson C.L., Slish M.L., Rhoades-Kerswill S., Mills A.C., Judah M.R. (2013). An Examination of the Reciprocal Relationship between Avoidance Coping and Symptoms of Anxiety and Depression. J. Soc. Clin. Psychol..

[73] Compas B.E., Desjardins L., Vannatta K., Young-Saleme T., Rodriguez E.M., Dunn M., Bemis H., Snyder S., Gerhardt C.A. (2014). Children and Adolescents Coping with Cancer: Self-and Parent Reports of Coping and Anxiety/Depression. Health Psychol..

[74] Xie J., Liu M., Zhong Z., Zhang Q., Zhou J., Wang L., Ma K., Ding S., Zhang X., Sun Q. (2020). Relationships among Character Strengths, Self-Efficacy, Social Support, Depression, and Psychological Well-Being of Hospital Nurses. Asian Nurs. Res..

[75] Mohd T.A.M.T., Yunus R.M., Hairi F., Hairi N.N., Choo W.Y. (2019). Social Support and Depression among Community Dwelling Older Adults in Asia: A Systematic Review. BMJ Open.

[76] Poh Chua S., Xiang Yi T., Chin Choo G.Y., Chee Seng T., Tjin Ai J.T., Sew Kim L., Lokithasan K.A. (2021). Cyber-Victimization among Adolescents: Its Relationships with Primary Appraisal and Coping Strategies. Vulnerable Child. Youth Stud..

[77] Guo S. (2021). A Comparison of Traditional Victims, Cyber Victims, Traditional-Cyber Victims, and Uninvolved Adolescents: A Social-Ecological Framework. Child Youth Care Forum J. Res. Pract. Child. Serv..

[78] McLoughlin L.T., Spears B.A., Taddeo C.M., Hermens D.F. (2019). Remaining Connected in the Face of Cyberbullying: Why Social Connectedness Is Important for Mental Health. Psychol. Sch..

[79] Chu X.-W., Fan C.-Y., Liu Q.-Q., Zhou Z.-K. (2018). Cyberbullying Victimization and Symptoms of Depression and Anxiety among Chinese Adolescents: Examining Hopelessness as a Mediator and Self-Compassion as a Moderator. Comput. Hum. Behav..

[80] Shaughnessy J., Zechmeister E., Zechmeister J. (2015). Research Methods in Psychology.

[81] Chun J., Lee J., Kim J., Lee S. (2020). An International Systematic Review of Cyberbullying Measurements. Comput. Hum. Behav..

